# Exploring a New Adaptive Routing Based on the Dijkstra Algorithm in Optical Networks-on-Chip

**DOI:** 10.3390/mi12010054

**Published:** 2021-01-05

**Authors:** Yan-Li Zheng, Ting-Ting Song, Jun-Xiong Chai, Xiao-Ping Yang, Meng-Meng Yu, Yun-Chao Zhu, Yong Liu, Yi-Yuan Xie

**Affiliations:** 1 School of Electronic and Information Engineering, Southwest University, Chongqing 400715, China; zhengyanlis@email.swu.edu.cn (Y.-L.Z.); ttsong_53@163.com (T.-T.S.); jxchai@email.swu.edu.cn (J.-X.C.); xiaoping_415@foxmail.com (X.-P.Y.); ymmsunraise@163.com (M.-M.Y.); 15668910386@163.com (Y.-C.Z.); 2 School of Optoelectronic Information, University of Electronic Science and Technology of China, Chengdu 611731, China; yongliu@uestc.edu.cn

**Keywords:** optical networks-on-chip, Dijkstra algorithm, transmission loss, optimized power control

## Abstract

The photoelectric hybrid network has been proposed to achieve the ultrahigh bandwidth, lower delay, and less power consumption for chip multiprocessor (CMP) systems. However, a large number of optical elements used in optical networks-on-chip (ONoCs) generate high transmission loss which will influence network performance severely and increase power consumption. In this paper, the Dijkstra algorithm is adopted to realize adaptive routing with minimum transmission loss of link and reduce the output power of the link transmitter in mesh-based ONoCs. The numerical simulation results demonstrate that the transmission loss of a link in optimized power control based on the Dijkstra algorithm could be maximally reduced compared with traditional power control based on the dimensional routing algorithm. Additionally, it has a greater advantage in saving the average output power of optical transmitter compared to the adaptive power control in previous studies, while the network size expands. With the aid of simulation software OPNET, the network performance simulations in an optimized network revealed that the end-to-end (ETE) latency and throughput are not vastly reduced in regard to a traditional network. Hence, the optimized power control proposed in this paper can greatly reduce the power consumption of s network without having a big impact on network performance.

## 1. Introduction

With constantly improving manufacturing and integration of on-chip technology involving complementary metal oxide semiconductors (CMOS), the number of processing cores on a die is increasing dramatically. Therefore, the traditional Networks cannot meet the demands of larger bandwidth and lower latency. Optical networks-on-chip (ONoCs) are being equipped with higher bandwidth, lower delay, and higher energy efficiency so that they can become an effective solution to chip multiprocessor (CMP) systems [1,2,3,4]. The optical router is the crucial component of the ONoC communication system. It consists of basic optical switching elements, a waveguide crossings, and an optical terminal. The switching elements, waveguide crossings, and propagation loss inside the optical router will cause transmission loss. Unfortunately, larger transmission loss will lead to more power consumption and greatly limit the expansion of ONoCs [5].

Undoubtedly, reducing power consumption attaches great significance to improving network performance. Nowadays, the studies of reducing power consumption are divided into three aspects: changing the nanophotonic architecture, optimizing the routing algorithm, and optimizing the circuit. The nanophotonic architecture can be optimized in order to minimize the laser power consumption. In previous studies, a novel fat-tree floorplan [6] and a ring-based packet-switched optical network-on-chip (RPNoC) [7] were proposed to reduce the number of hops of routing path so as to yield lower energy consumption. Recently, an optimized routing algorithm based on Gaussian-based ONoCs was proposed to improve the optical signal-to-noise ratio (OSNR) [8]. The length-optimized-routing-protocol (LORP) [1] was designed to select the preferred option with the smallest number of hops in a virtual cluster in mesh. A new algorithm [9] to select the routing path with the lowest power consumption was proposed to reduce the power loss and raise the OSNR. It improves the transmission quality of the optical signal and lowers energy consumption. The external laser power was reduced by optimizing the circuit in previous studies [10,11,12]. Runtime power management techniques have been proposed to reduce the magnitude of laser power consumption by tuning the network in response to actual application characteristics [13,14]. The traditional power control method is to compare the maximum power of each node and select the maximum power value among them to allocate it to each node. Based on this, a method based on time division multiplexing was proposed to optimized it and save on total laser power in [15].

It is known that adaptive power control (APC) could save energy and reduce power consumption. The APC was firstly proposed to decrease the total power consumption, and then the APC was applied in [16,17,18,19,20]. In [20], the overall SNR was improved by the combination of APC and the new clustering design. The APC adjusts the transmission loss of each link according to the ratio between the signal transmission loss and the maximum transmission loss. However, considering that only the link with the largest transmission loss makes full use of the power in APC, there is still more power wasted in other links of the network. Focusing on this issue, we used the Dijkstra algorithm to realize a new adaptive routing method which achieves minimum transmission loss for every link in mesh-based ONoCs. In this paper, the Dijkstra algorithm takes the port-to-port transmission loss of the router as the weight value between neighboring routers and selects the next route by comparing the weight values. Considering that the Dijkstra algorithm is globally optimal, it can select the routing path with the minimum transmission loss of each link in a network. We evaluated the power relationship between the optical transmitter and the optical receiver during the transmission of an optical signal on a silicon photonic link. The output power of the optical transmitter on a silicon photonic link depends on the transmission loss of the optical switching device, the transmission loss of the waveguides, and the sensitivity of the receiver. On the premise of keeping the receiver’s sensitivity unchanged, the power consumption can be decreased by reducing the link transmission loss. The implementation of optimized power control based on the Dijkstra algorithm can achieve the minimum link transmission loss and greatly reduce the power consumption of the whole network. By the transmission loss analysis model and numerical simulations, we found when the network sizes are 5×5, 6×6, 7×7, and 8×8, the optimized average output power can reach −15.6376, −15.3482, −15.1276, and −14.9548 dBm respectively. Regarding network performance, the traditional and optimized networks reached saturation when the offered loads were 0.1 and 0.08, and 0.06 and 0.04, respectively. Compared with the traditional dimensional routing, the end-to-end (ETE) delay and throughput obtained by OPNET are not significantly reduced, which indicates that the optimized power control is feasible.

## 2. Internal Insertion Loss Analysis of ONoCs

**Figure 1:**
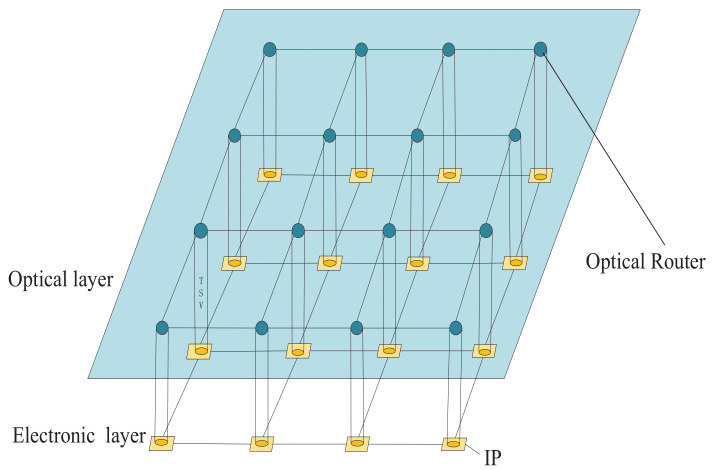
Hybrid optical mesh network-on-chip (NoC) construction.

The traditional network uses an electrical signal to transmit information. It has small bandwidth and needs a lot of energy so as to hardly meet the communication demand. Compared with electronic interconnections, optical interconnects can not only meet the future system bandwidth requirements, but also show the advantages of lower latency and smaller power consumption. Electronic interconnections are fit for short distance communication, whereas optical interconnections do well in long distance communication. Therefore, in order to make full use of the advantages of optical interconnections and electronic interconnections, the hybrid optical mesh NoC [21] was proposed. It consists of an electrical connection layer, an optical connection layer, and a connection layer through-silicon-vias (TSVs) between the two layers. TSVs are used to transmit the signals from the electronic layer to the optical layer. In this paper, we use the hybrid optical mesh NoC shown in Figure 1. Besides, the electrical interconnection layer and the optical interconnection layer work together. The former contributes to transmitting control information to links and the later is mainly aimed at transmitting sensor data.

### 2.1. Basic Optical Switching Elements (BOSEs)

**Figure 2:**
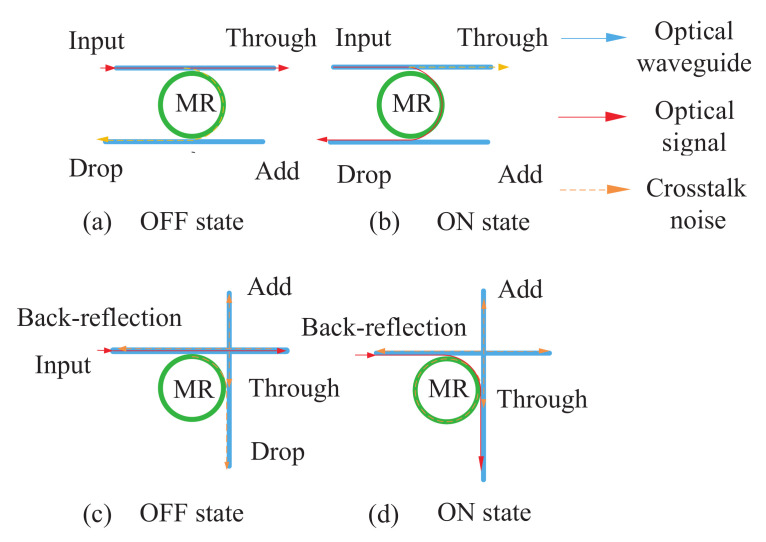
Basic 1 × 2 switching elements using microresonators.

An optical router consists of waveguide crossings, waveguides, optical terminators, and BOSEs. BOSEs are composed of waveguides and microresonators [10]. There are two basic 1×2 optical switching elements which are the crossing switching element (CSE) and the parallel switching element (PSE). Besides, the CSE consists of two crossing waveguides and a microring resonator adjacently located between two crossing waveguides shown in Figure 2a,b. The PSE is composed of two parallel waveguides and a microring resonator locating between two parallel waveguides shown in Figure 2c,d.

As shown in Figure 2, PSE and CSE have two states, namely on state and off state. If the wavelength of the optical signal is different from the resonant wavelength, the optical signal will be output to the through port rather than being coupled into the ring. This state is called off state, which is shown in Figure 2a,c. The on states of PSE and CSE are shown in Figure 2b,d. When the wavelength of the optical signal is equal to the resonant wavelength, the optical signal is transmitted from the input port to be coupled with the ring and directed to the drop port. We ignore the crosstalk noise on the add port of the PSE in this analysis according to a previous study [10]. In order to understand the notations in the formula, Table 1 lists the meanings of the relevant notation. The parameters in Table 1 are used for performance evaluation. In addition, the insertion loss is the output power coefficient of optical switching elements, while the transmission loss is the loss coefficient of the optical switching elements in this paper.

The output powers of the through port and drop port as functions of input optical power could be calculated based on (1) and (2) for the off state and (3) and (4) for the on state.

(1) $$P_{T,off}=P_{in}L_{p,off}$$

(2) $$P_{D,off}=P_{in}K_{p,off}$$

(3) $$P_{T,on}=P_{in}L_{p,on}$$

(4) $$P_{D,on}=P_{in}K_{p,on}$$

When two optical signals pass through the crossing at the same time in CSE, the optical signals interfere with each other and crosstalk occurs. At the same time, a portion of the light will be reflected back to the input port. When CSE is in the on state, the output power values of different ports are calculated as (5)–(8) according to previous studies [10]. When the CSE is in the off state, the optical signal enters from the input port and is coupled to the ring, and then is directed to the drop port. The formulae for calculating the output power at different ports are shown in (9)–(12).

(5) $$P_{D,on}=P_{in}L_{c,on}$$

(6) $$P_{T,on}=P_{in}K_{p,on}\left(L_{c}\left(1+K_{c}L_{p,on}\right)+K_{r}L_{p,on}K_{c}\right)$$

(7) $$P_{A,on}=P_{in}K_{p,on}\left(K_{c}\left(1+K_{c}L_{p,on}\right)+K_{r}L_{p,on}L_{c}\right)$$

(8) $$P_{R,on}=P_{in}K_{p,on}K_{c}$$

(9) $$P_{T,off}=P_{in}L_{c,off}$$

(10) $$P_{D,off}=P_{in}\left(K_{p,off}+L_{p,off}^{2}K_{c}\right)$$

(11) $$P_{A,off}=P_{in}K_{p}L_{p,off}^{2}$$

(12) $$P_{R,off}=P_{in}K_{r}L_{p,off}^{2}$$

### 2.2. Five-Port Non-Blocking Router Model

**Figure 3:**
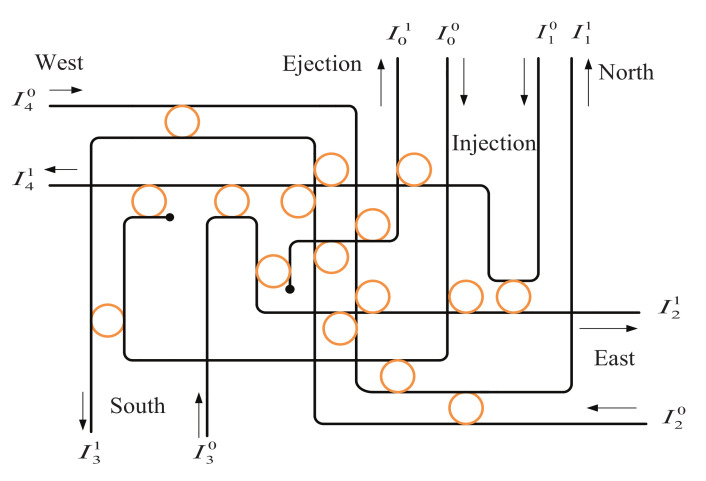
Five-port non-blocking router model.

The optical router plays an important role in ONoCs. The five-port non-blocking router [24] has fewer microring resonators (MRRs) and crossings, which means less transmission loss to some extent. The five-port non-blocking router used in this paper is shown in Figure 3; $I_{i}^{j}$ = $I_{i}^{0}$ is the input port of the *i*th port and $I_{i}^{j}$ = $I_{i}^{1}$ is the output port of the *i*th port. Meanwhile, when *i* = 0, 1, 2, 3, and 4, they represent ejection, north, east, south, and west ports.

When an optical signal is transmitted from the *i*th port to the *j*th port in the router, the transmission loss can be defined as $L_{i,j}\left(x,y\right)$ in (13). The propagation loss is integrated at the network level into our general optical router model [25]. The CSE, PSE, waveguide crossings, and propagation loss $L_{P}$ will cause switching loss $S_{Li,j}\left(x,y\right)$. $W_{l_{i,j}}\left(x,y\right)$ represents the waveguide length from the *i*th input port to the *j*th output port inside the router. When j=0, the output port is ejection. When j≠0, the calculation of $L_{i,j}\left(x,y\right)$ also needs to take the waveguide transmission loss between the current router and the next router into account. In (14), hop length *D* can be attained based on chip size *S* and network size M×N. If the input optical power $P_{i}^{0}\left(x,y\right)$ is determined, then the output power $P_{i,j(x,y)}$ from the *i*th port to the *j*th port of the router can be calculated as the formula shown in (15).

(13) $$L_{i,j}\left(x,y\right)=\left\{\begin{array}{cc} 1-S_{L_{i,j}}\left(x,y\right)L_{P}^{W_{l_{i,j}}\left(x,y\right)} & j=0\\ 1-S_{L_{i,j}}\left(x,y\right)L_{P}^{W_{l_{i,j}}\left(x,y\right)+D} & j\neq 0 \end{array}\right.$$

(14) $$D\approx \sqrt{\frac{S}{M\times N}}$$

(15) $$P_{i,j}\left(x,y\right)=P_{i}^{0}\left(x,y\right)\left(1-L_{i,j}\left(x,y\right)\right)$$

### 2.3. Adaptive Power Control Model

The output power of the optical transmitter on the link is determined on the transmission loss of the optical switching device, the transmission loss of the waveguide, and the sensitivity of the receiver. According to [26], the optical signal power received from the receiver should not be less than the sensitivity of the receiver. As is shown in (16), $P_{TX}$ is the output power of the link transmitter, $L_{SW}$ is the optical transmission loss of the optical switching device, $L_{WG}$ is the optical transmission loss of the waveguides, and $S_{RX}$ is the sensitivity of the receiver. In [27], the high-speed optical receiver used was manufactured via Ge waveguide optical detector integrated into a CMOS process. It could achieve the receiver sensitivity of −14.2 dBm operating at 1550 nm and 10 Gbps. In (17), $P_{TX}$ can be obtained by derivation of (16). In this paper, we optimize the selection of routing path based on the Dijkstra algorithm from the source node to destination node so as to achieve the minimum of the sum of $L_{SW}$ and $L_{WG}$. Therefore, on the basis that the receiver sensitivity remains the same, the output power $P_{TX}$ of the link transmitter should be the minimum.

(16) $$P_{TX}-L_{SW}-L_{WG}\geq S_{RX}$$

(17) $$P_{TX}\geq L_{SW}+L_{WG}+S_{RX}$$

The power of *s* node is allocated in terms of the maximum link transmission loss which is obtained from node *s* to all destination nodes [16]. The maximum transmission loss of node *s* can be calculated as (18), where $IL_{max}^{s}$ represents the maximum transmission loss of node *s*; the link transmission loss from source node *s* to destination node *d* is denoted by $IL_{sd}$. In traditional power control, the power of each node is equally distributed, which is composed of the largest value among the maximum transmission loss $IL_{max}^{s}$ of all nodes. The maximum transmission loss MaxIL among all of the $IL_{max}^{s}$ is shown in (19).

(18) $$IL_{max}^{s}=max\left(IL_{sd}\right)\;for\left(d\neq s\right),d\in \left(0,N\right)$$

(19) $$MaxIL=max\left(IL_{max}^{s}\right),s\in \left(0,N\right)$$

In traditional power control [16], each link is allocated the maximum transmission loss. Thus, the transmission losses of many links will be redundant when the actual transmission loss is less than the maximum transmission loss. To solve this problem, the APC technology is applied for link power distribution [20]. For example, in terms of links A, B, and C, we treat $L_{A}$, $L_{B}$, and $L_{C}$ as the maximum transmission losses of the three links respectively. After comparison, we can obtain the maximum transmission loss (i.e., $L_{A}$) of the three links. The input power of link B and link C can be adjusted according to link A, and the calculations are shown in (20)–(24).

(20) $$P_{output-A}=P_{Input-A}\left(1-L_{A}\right)$$

(21) $$P_{output-B}=P_{Input-B}\left(1-L_{B}\right)$$

(22) $$P_{output-C}=P_{Input-C}\left(1-L_{C}\right)$$

(23) $$P_{Input-B}=\frac{1-L_{A}}{1-L_{B}}P_{Input-A}$$

(24) $$P_{Input-C}=\frac{1-L_{A}}{1-L_{C}}P_{Input-A}$$

$P_{Input-A}$, $P_{Input-B}$, and $P_{Input-C}$ are the input power of links A, B, and C respectively, while $P_{output-A}$, $P_{output-B}$, and $P_{output-C}$ are the output power of links A, B, and C respectively. According to the above equations, if the link transmission loss is closer to the maximum link transmission loss, the input power of the corresponding link needs to be larger. Considering that the transmission losses of link B and C are always less than $L_{A}$, the input power of link B and C will always less than link A, which has the maximum transmission loss. In spite of APC being able to reduce power consumption to some extent, it still can not solve the problem of the global minimum of network power usage. The Dijkstra algorithm is adopted to select the routing path with minimal transmission loss on account that it has the characteristics of global optimization. Therefore, the output power of the link transmitter can be the minimal on the basis that the receiver sensitivity remains constant.

## 3. Adaptive Power Control of ONoCs Based on the Dijkstra Algorithm

### 3.1. The Dijkstra Algorithm’s Principles

The Dijkstra algorithm is the shortest path algorithm from one vertice to the other vertices. Considering that it is suitable for undirected and directed graphs with positive weights, we take the port-to-port transmission loss of router in ONoCs as weights of the Dijkstra algorithm. The principles of the Dijkstra algorithm are as follows: Firstly, set S contains only the source nodes and set U contains all the remaining nodes. The distances in set U represent the distances between the starting vertice and other vertices. Secondly, it compares the stored distances and finds the node which is the closest to the source node, and then adds it to set S. At the same time, it removes this node from set U and updates the distance from each vertex in U to the current vertex. Thirdly, it traverses again to compare whether the distances thanks to choosing the shortest point as a transit point are more closer or not. If so, it just updates the distance; otherwise, it does not update. Ultimately, it repeats the previous second and third steps until all the vertices are traversed in U.

In a previous study [28], the shortest path first (SPF) algorithm was applied to select the path with the lowest bit error rate among all shortest available paths. The shortest distance from the starting node to all the nodes could be stored after traversing. The shortest spanning tree algorithm can choose the path with the least sum of weights which is from the starting vertice to other vertices. The Dijkstra algorithm could attain the shortest path of weights from the fixed starting vertice to the fixed ending vertice. Therefore, the Dijkstra algorithm is more suitable to optimized routing paths for network architectures when the source and destination nodes are known. The Dijkstra algorithm is described in detail in Algorithm 1. In this paper, the port-to-port transmission loss of a router serves as the weight of the selection of routing path; the path with minimum transmission loss from the source node to the destination node will be determined by the Dijkstra algorithm.

### 3.2. Network Architecture

**Figure 4:**
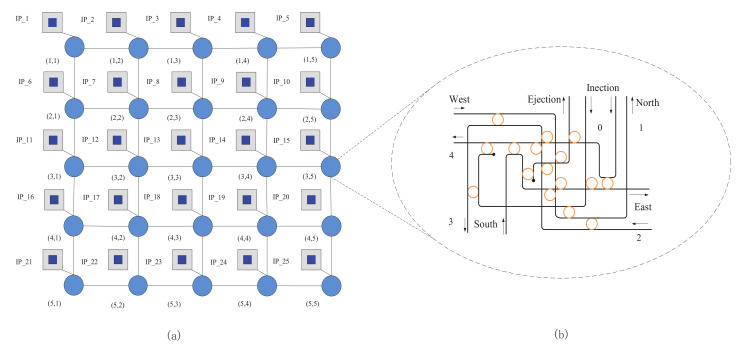
(**a**) The 5×5 mesh model based ONoCs; (**b**) The internal structure of the router.

Based on the 5×5 mesh-based ONoCs shown in Figure 4, we analyze how to select the routing path using the Dijkstra algorithm as follows. The 5×5 mesh model based ONoCs is shown in Figure 4a, and the internal structure of the router is shown in Figure 4b. We use a two-dimensional coordinate system to denote the position of the router in the 5×5 mesh-based ONoCs shown in Figure 4a. For example, the coordinate of router connected to core $IP_{-}1$ is expressed as (1, 1). $L_{i,j}\left(x,y\right)$ represents the transmission loss from the *i*th output port to the *j*th input port in router (x,y). In the Dijkstra algorithm, it is used as the weight value between the current router and the next-hop router which the *j*th port of the current router will be connected to. In multiple pairs of source nodes in concurrent communication with mesh-based ONoCs, the coordinates of source node and destination node can be divided into four types. The destination node is in the upper left, upper right, lower left, or lower right of the source node. We use a two-dimensional matrix to store all possible port transmission losses of the router in Figure 4b. After obtaining the packet with the coordinate information of the destination node, the corresponding weights of router will be updated according to the transmission loss stored in the two-dimensional matrix. Moreover, the Dijkstra algorithm is used to compare the port-to-port transmission loss of the current router and determine the next hop router. For example, when processor core $IP_{-}1$ communicates with processor core $IP_{-}8$, the optical signal will be transmitted from $IP_{-}1$ to router (1,1) through the 0th input port of router (1,1). We choose $L_{0,2}\left(1,1\right)$ as the transmission loss between router (1,1) and router (1,2), then treat $L_{0,3}\left(1,1\right)$ as the transmission loss between router (1,1) and router (2,1). Firstly, compare $L_{0,2}\left(1,1\right)$ and $L_{0,3}\left(1,1\right)$. In terms of the internal structure of router used in this paper, $L_{0,2}\left(1,1\right)$ is smaller; the optical signal will be transmitted from the 2nd output port of router (1,1) to the 4th input port of router (1,2). Secondly, compare the transmission loss $L_{4,3}\left(1,2\right)$ between the processor core router (1,2) and router (2,2) and the transmission loss $L_{4,2}\left(1,2\right)$ between processor core router (1,2) and router (1,3). As $L_{4,3}\left(1,2\right)$ is smaller, the optical signal will be transmitted from the 3rd output port of router (1,2) to the 1st input port of router (2,2). More importantly, the Dijkstra algorithm will calculate and compare between $L_{0,2}\left(1,1\right)$ + $L_{4,3}\left(1,2\right)$ and $L_{0,3}\left(1,1\right)$ + $L_{1,2}\left(2,1\right)$ to determine whether the routing path achieves the minimum transmission loss or not. Finally, the optical signal will be transmitted from the 2nd output port of router (2,2) to the 4th input port of router (2,3). The optical signal passes from the 0th output port of the router (2,3) to processor core $IP_{-}8$ to complete this communication.

Ultimately, when processor core $IP_{-}1$ communicates with processor core $IP_{-}8$, the routing path selected by the above algorithm is: $IP_{-}1$-router (1,1)-router (1,2)-router (2,2)-router (2,3)-$IP_{-}8$.

**Figure 5:**
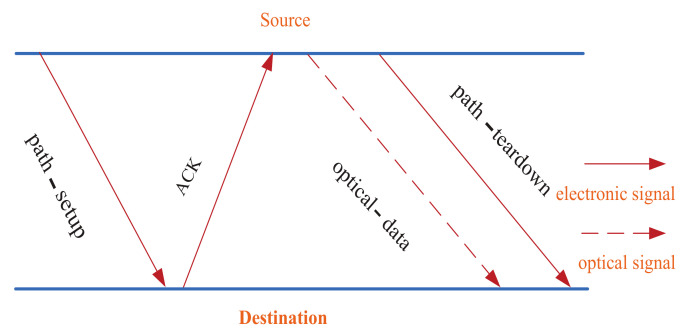
Mechanism diagram of optical circuit switching.

The control information and data are transmitted through the electrical link in the traditional electronic interconnection network, whose packet switching mechanisms are circuit switching, packet switching, wormhole switching, etc. The opto-electronic interconnection network on the chip is composed of the combination between the electronic interconnection layer and the optical interconnection layer. Considering that there is no effective buffering technique for optical signal in ONocs so far, the circuit has great difficulty in reserving the routing path. Alleviating this intractable problem, most of the existing active ONoCs depend on the optical circuit switching (OCS) mechanism [1], as shown in Figure 5.
**Algorithm 1:** Routing algorithm based on the Dijkstra algorithm
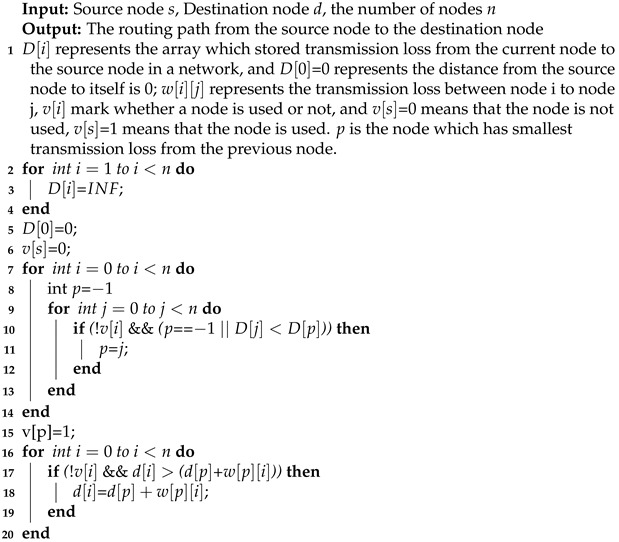


The optical layer and the electrical layer work coordinately in the OCS mechanism. The electrical layer transmits link controlling information and the optical network layer transmits data. Before transmitting the optical signal, it is necessary to establish the reserved path between the source node and destination node, which is achieved by the path-setup, ACK, and path-teardown packets from the electrical control layer. In the setup stage of optical path, if there are other communication requests in the occupied phase of the router port in a node, the path-setup packet will be set to cache on this node only if the port is released. The transmission latency of optical signal depends only on the group velocity of light in the silicon based waveguide [29,30] so that it is very short. Therefore, the latency of the setup path with electronic singal is crucial for the communication latency.

## 4. Evaluation and Discussion

On the premise of not sacrificing the network’s performance, optimized power control based on the Dijkstra algorithm in ONoCs has obvious advantages in saving power over adaptive power control in previous studies. Section 4.1 indicates the reduction of the transmission loss in the optimized mesh-based ONoCs is larger than that in the traditional mesh-based ONoCs. The average output power of the transmitter of optimized power control by using the Dijkstra Algorithm is better than that in the adaptive power control and traditional power control. Section 4.2 shows the comparison of network performance between an optimized network based on the Dijkstra algorithm and a traditional network based on the dimensional routing algorithm under different network sizes.

### 4.1. Transmission Loss and Average Output Power of Transmitter

In mesh-based ONoCs, transmission loss based on the Dijkstra algorithm is compared with that based on traditional dimensional routing with the aid of MATLAB software. The average transmission loss ${\bar{L}}_{\left(s,d\right)}$ of all links from the source node to the other nodes is considered as the transmission loss evaluation value of network; the formula is shown in (25). Source node, destination node, and total number of nodes in the network are respectively represented by *s*, *d*, and *n*. $L_{\left(s,d\right)}$ represents the link transmission loss from source node *s* to destination node *d*. Here we select node (1,1) as the source node for analysis.

(25) $${\bar{L}}_{\left(s,d\right)}=\frac{\sum_{s=1}^{n}\sum_{d=1}^{n}L_{\left(s,d\right)}}{n}$$

**Figure 6:**
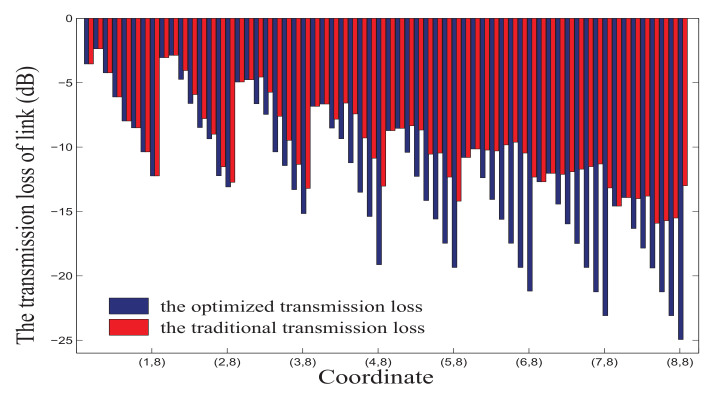
The transmission loss in M×N mesh-based ONoCs.

Under the condition that the network size is 8×8, the transmission losses of the optimized link and traditional link from the source node (1,1) to other nodes are shown in Figure 6. It is clearly visible that the optimized link has less link transmission loss than the traditional link.

**Figure 7:**
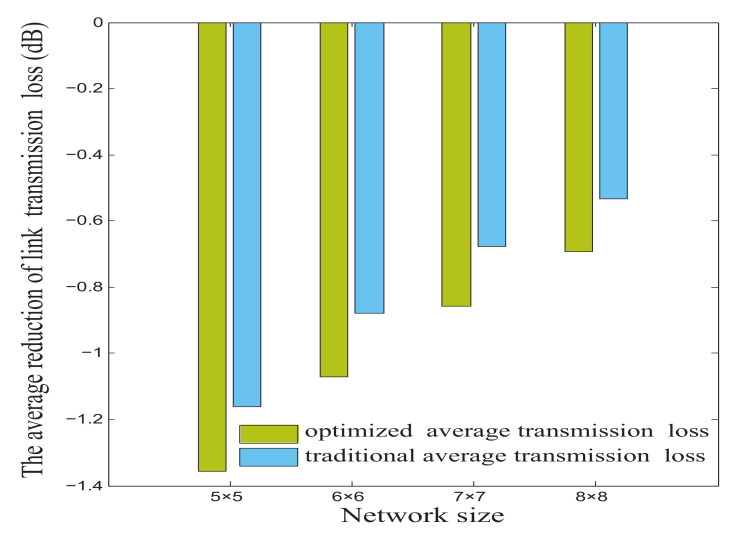
The average reduction of link transmission loss in M×N mesh-based ONoCs.

As shown in Figure 7, when network sizes are 5×5, 6×6, 7×7, and 8×8, the optimized average link transmission losses are 0.193, 0.194, 0.183, and 0.161 dB, respectively—lower than the traditional average link transmission loss.

The average output power of the transmitter in network links is ${\bar{P}}_{T(x,y)}$ and the formula is shown in (26). *M* and *N* represent network sizes, and (x,y) represents the location of source node in network link. $P_{T(x,y)}$ represents the output power required by the optical transmitter of the source node in a network link.

(26) $${\bar{P}}_{T(x,y)}=\frac{\sum_{y=1}^{M}\sum_{x=1}^{N}P_{T(x,y)}}{M\times N}$$

**Figure 8:**
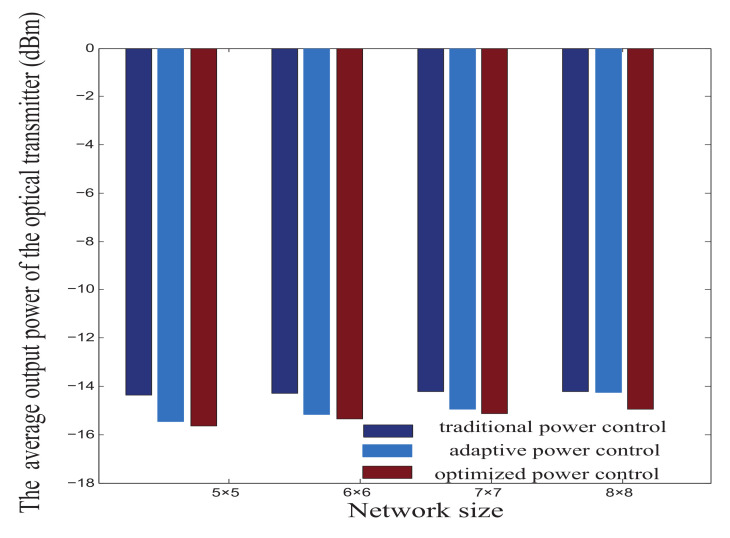
The average output power of the optical transmitter in M×N mesh-based ONoCs.

Figure 8 shows the average output power of the optical transmitter under traditional power control, adaptive power control, and optimized power control when the network sizes are 5×5, 6×6, 7×7, and 8×8. The transmission losses caused by increasing the numbers of optical switch devices and waveguides constitute the main reason that the output power of the transmitter increases with the expansion of the network. From Figure 8, it can be concluded that the average output power of the transmitter under the traditional power control is the highest, followed by the adaptive power control, and the optimized power control has the lowest output power. Hence, the optimized power control could realize the minimum output power of transmitter of links.

End-to-end (ETE) delay and throughput are important indicators for evaluating network performance. We constructed a simulation of an optimized network and a traditional network with the aid of OPNET simulation software, which is based on discrete event scheduling works. In order to better analyze the network performance under different network sizes, we implemented four network sizes for analysis: 5×5, 6×6, 7×7, and 8×8.

**Figure 9:**
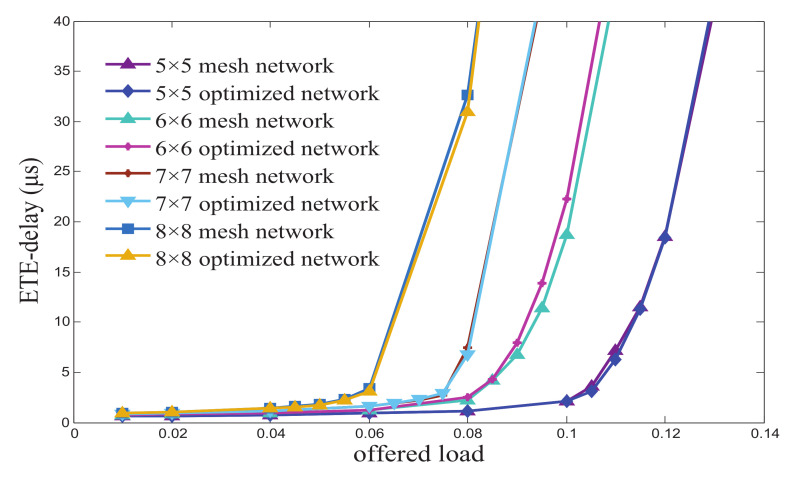
The ETE-delay in M×N mesh-based ONoCs.

Figure 9 demonstrates that when the packet size is 1024 bits, the ETE-delay of the traditional network is a bit lower than that in the optimized network. What is more, with the expansion of network sizes, network congestion is serious, resulting in the vastly increasing ETE-delay. When the network size is 5×5, 6×6, 7×7, or 8×8, the offered loads for the traditional network and optimized network reach saturation—0.1, 0.08, 0.06, and 0.04 respectively.

### 4.2. Network Performance Evaluation

**Figure 10:**
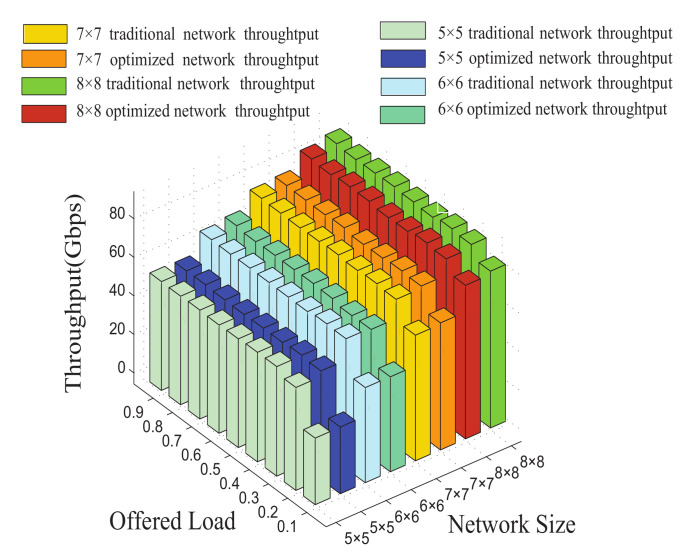
The network throughput in M×N mesh-based ONoCs.

It is shown for the network throughput in Figure 10. Under the same load, the network based on the Dijkstra algorithm has almost the same throughput compared with the network based on the dimensional-order routing algorithm with the increasing of the network size. Furthermore, as the load is increasing, the throughput of the traditional network and the optimized network gradually increase to saturation.

## 5. Conclusions

In conclusion, a new adaptive routing algorithm based on the Dijkstra algorithm realizes the selection of routing path with minimum transmission loss in the paper. Taking mesh-based ONoCs as an example, the optimized path based on the Dijkatra algorithm can achieve the minimum link transmission loss, and the average link transmission loss is significantly reduced compared with the traditional dimensional routing path under different network sizes. At the same time, compared with the traditional power control and adaptive power control, the optimized power-control-based Dijkstra algorithm has a significant advantage in reducing power consumption. In terms of network performance, we verified the feasibility of this adaptive routing algorithm to realize the selection of minimum transmission loss routing path with the aid of simulation software. According to the simulation results, the ETE-delay of the optimized network is a little larger than that in the traditional network. Moreover, with the expansion of the network scale, the throughput was almost the same as that in the traditional network based on dimensional routing algorithm. Therefore, optimized power control could reduce power consumption without greatly reducing network performance. In the future, the performance of minimum transmission loss routing path selection based on the Dijkstra algorithm in different network structures needs to be studied.

## Figures and Tables

**Table 1:** Power loss factors of a basic optical device.

Parameter	Notation	Value	Reference
Crossing loss	$L_{c}$	−0.04 dB	[22]
Power loss per PSE in ON state	$L_{p,on}$	−0.5 dB	[23]
Power loss per PSE in ON state	$L_{p,off}$	−0.005 dB	[23]
Crosstalk coefficient per PSE in OFF state	$k_{p,off}$	−45 dB	[10]
Crosstalk coefficient per PSE in ON state	$k_{p,off}$	−25 dB	[10]
Power loss per CSE in OFF state	$L_{c,off}$	−0.04 dB/90${}^{\circ }$	[10]
Power loss per CSE in ON state	$L_{c,on}$	−0.5 dB/90${}^{\circ }$	[10]
Crossing crosstalk coefficient	$k_{c}$	−38.5 dB/90${}^{\circ }$	[10]
